# Camelina Oil Supplementation Improves Bone Parameters in Ovariectomized Rats

**DOI:** 10.3390/ani11051343

**Published:** 2021-05-09

**Authors:** Iwona Puzio, Dorota Graboś, Marek Bieńko, Radosław P. Radzki, Aneta Nowakiewicz, Urszula Kosior-Korzecka

**Affiliations:** 1 Department of Animal Physiology, Faculty of Veterinary Medicine, University of Life Sciences in Lublin, Akademicka St. 12, 20-950 Lublin, Poland; dorotagrabos@interia.pl (D.G.); marek.bienko@up.lublin.pl (M.B.); radoslaw.radzki@up.lublin.pl (R.P.R.); 2 Department of Preclinical Veterinary Sciences, Faculty of Veterinary Medicine, University of Life Sciences in Lublin, Akademicka St. 12, 20-950 Lublin, Poland; aneta.nowakiewicz@up.lublin.pl (A.N.); urszula.korzecka@up.lublin.pl (U.K.-K.)

**Keywords:** camelina oil, bone, polyunsaturated fatty acids, rat, ovariectomy

## Abstract

**Simple Summary:**

Supplementation of ovariectomized rats with *Camelina sativa* oil, which is rich in polyunsaturated fatty acids (PUFA), especially n-3 family fatty acids, can be an effective way to improve bone parameters. Administration of 5 g/kg body weight and 9 g/kg body weight of camelina oil to rats suppressed a decrease in densitometric, tomographic, and strength parameters of femurs and an increase in the serum level of the C-terminal telopeptide of type I collagen caused by estrogen deficiency. Furthermore, ovariectomized rats receiving camelina oil were characterized by a high level of osteocalcin.

**Abstract:**

The aim of the present study was to determine the effect of administration of *Camelina sativa* oil (CO) as a source of polyunsaturated fatty acids (PUFA) on bone parameters in ovariectomized rats (OVX). Overall, 40 10-week-old healthy female Wistar rats were divided into 4 groups with 10 animals in each. Rats in the control group (SHO) were subjected to a sham operation, whereas experimental rats (OVX) were ovariectomized. After a 7-day recovery period, the SHO the rats received orally 1 mL of physiological saline for the next 6 weeks. The OVX rats received orally 1 mL of physiological saline (OVX-PhS), 5 g/kg BW (OVX-CO5), or 9 g/kg BW (OVX-CO9) of camelina oil. The use of camelina oil had a significant effect on body weight, lean mass, and fat mass. The camelina oil administration suppressed the decrease in the values of some densitometric, tomographic, and mechanical parameters of femur caused by estrogen deficiency. The CO treatment increased significantly the serum level of osteocalcin and decreased the serum level of C-terminal telopeptide of type I collagen in the OVX rats. In conclusion, camelina oil exerts a positive osteotropic effect by inhibiting ovariectomy-induced adverse changes in bones. Camelina oil supplementation can be used as an efficient method for improving bone health in a disturbed state. However, further research must be carried out on other animal species supplemented with the oil.

## 1. Introduction

Pathological bone and cartilage changes are an increasing problem in both livestock and companion animals. The health conditions include osteopenia with different etiologies, osteoporosis, osteochondrosis, rickets, and osteoarthritis [1,2,3,4]. In both groups of animals, they cause movement disorders and pain and reduce the quality of their life. In the case of pigs and poultry, they lead to production losses.

Hence, to improve the health of bones and minimize production losses, intensive studies are conducted to identify factors that can act preventively or therapeutically in bone diseases. One of the possibilities is the use of botanical compounds. In recent years, oilseeds and plant oils have aroused great interest. They are a source of n-3 and n-6 polyunsaturated fatty acids (PUFA), which have received considerable attention for their role in mitigation of bone disorders. Most PUFAs from the n-3 and n-6 families are not synthesized by mammals. Therefore, their precursors, i.e., such essential acids as α-linolenic acid (ALA, 18:3 n-3) and linoleic acid (LA, 18:2 n-6), must be supplied with food. The involvement of elongases and desaturases leads to formation of eicosapentaenoic acid (EPA, 20:5 n-3), docosahexaenoic acid (DHA, 22:6 n-3), and arachidonic acid (AA, 20:4 n-6). EPA and AA are substrates for production of eicosanoids; they compete for the same enzymes in the metabolic pathways [5,6]. Eicosanoids derived from n-3 PUFAs (3-series prostaglandins, 3-series thromboxanes, 5-series leukotrienes, E-series resolvins) generally have anti-inflammatory effects, whereas those produced from n-6 PUFAs (2-series prostaglandins, 2-series thromboxanes, 4-series leukotrienes) act as pro-inflammatory agents [7]. Eicosanoids derived from n-6 PUFAs influence the synthesis of pro-inflammatory cytokines, i.e., interleukin-1 (IL-1), interleukin-6 (IL-6), and tumor necrosis factor-α (TNF-α), which participate in the development of such skeletal disorders as osteopenia, osteoporosis, or arthritis [8].

There are many papers in the databases on the impact of various oils of plant and animal origin, as well as individual PUFAs on bone metabolism and properties in both animals and humans. A positive effect of seeds, flax oil, soybean oil, sunflower oil, sesame oil, and olive oil on bone tissue has been demonstrated [9,10,11,12,13,14]. However, there are also studies indicating a controversial impact of these oils [12,15,16,17].

In recent years, bioproducts from *Camelina sativa*, i.e., seeds, oil, and cake, have aroused considerable interest. *Camelina sativa*, a species from the Brassicaceae family also called false flax, gold-of-pleasure, wild flax, linseed dodder, German sesame, or Siberian oilseed, is one of the oldest cultivated plants. The history of cultivation of camelina goes back to the Bronze Age when this plant was used for consumption in Europe [18]. Camelina is a very fast growing annual or winter annual plant. The advantages of camelina include its relatively low habitat and agrotechnical requirements [19]. It grows in ruderal habitats and in arable fields. This plant resembles a weed: it has very low soil requirements and high drought tolerance. Camelina, therefore, offers lower production costs than other oilseed plants [20]. The increasing interest in the false flax in recent years is related to the high content of unsaturated fatty acids (around 90%) and PUFAs (50–60%), which are very valuable for animal and human health [21,22]. It has been found that the fatty acid composition of camelina oil is more favorable than the composition of a number of other plant oils, such as olive, flax, rapeseed, and sunflower oils. Camelina oil is characterized by high content of ALA (35–50%), lower content of LA (15–24%), and high content of tocopherols (about 800 mg/kg) and phenolic compounds, which results in greater oxidative stability compared to other oils containing large amounts of unsaturated acids [21,22,23,24,25,26,27]. Regardless of the fatty acids, the high nutritional value of camelina oils is also related to the content of other bioactive compounds such as tocopherols, carotenoids, and phytosterols, which lower the cholesterol level in blood, as well as optimal nutritional quality indices: the thrombogenicity index and the atherogenicity index [24,26,27]. Camelina oil also contains anti-nutritional substances like erucic acid and glucosinolates [21,22]. In an animal study, camelina bioproducts were found to influence positively the PUFAs content in eggs and meat of broiler chickens and rabbits, which is beneficial for human nutrition [28,29,30]. In quail, camelina was reported to prevent lipid oxidation without any adverse effect on performance and carcass characteristics [31]. Moreover, camelina oil in combination with conjugated linoleic acid improved bone densitometric parameters in broiler chickens [32]. However, there is no research on its effect on bone tissue when it is supplemented alone in mammals. Camelina oil also increased plasma n-3 fatty acid content and reduced plasma n-6 fatty acid content and serum triglyceride levels in pigs [33]. However, higher content of camelina bioproducts in food was found to cause a decrease in weight and feed intake, as well as poor feed conversion in animals [34,35]. With the properties mentioned above, camelina has been increasingly being appreciated for the last 20 years.

However, the influence of camelina bioproducts as a source of PUFAs on the metabolism of the skeletal system is very poorly known. Considering the prevalence of bone and joint disorders in animals and the composition of PUFAs in camelina oil, it can be assumed that supplementation with this product may positively influence bone tissue. Thus, the present study aimed to identify the potential osteotropic effects of camelina oil in female rats with experimental osteopenia induced by bilateral ovariectomy.

## 2. Materials and Methods

### 2.1. Animals and Experimental Design

In total, 40 10-wk-old healthy female Wistar rats (initial BW of approximately 220–240 g) were used in the study. Before the experiment, the rats were acclimatized to the new environmental conditions of the vivarium for 7 days. During the experiment, the rats were housed in a room with controlled temperature (22 ± 2 °C) and humidity (55 ± 10%) under a 12:12 h light–dark cycle with access to commercial diet for laboratory animals (Agropol, Motycz, Poland) and water at all times (except for a period of overnight fasting prior to the surgery and euthanasia). The rats were kept in plastic cages with sawdust bedding. On the day of the surgery, the rats were anaesthetized with an intramuscular injection of ketamine (Biowet-Puławy, Puławy, Poland), xylazine (SPOFA, Prague, Czech Republic), and atropinum sulphuricum (Polfa-Warszawa, Warsaw, Poland) at the doses of 10, 10, and 0.1 mg/kg of BW, respectively, and submitted to a sham operation (SHO; *n* = 10) or an ovariectomy (OVX) (*n* = 30). During the SHO operation, an incision was made in the abdominal wall, the viscera were repositioned manually, and the wound was sutured. In turn, the ovaries were removed during OVX. After the surgery, the rats were placed individually in plastic cages for a 7-day recovery period. No symptoms of postoperative complications were observed. No analgesics were administered to the rats during the postoperative period. Subsequently, the OVX rats were randomly divided into three groups receiving orally 1 mL of physiological saline (OVX-PhS) (*n* = 10) or camelina oil in the amount of 5 g/kg/BW (OVX-CO5; *n* = 10) or 9 g/kg/BW (OVX-CO9; *n* = 10) once a day for 6 weeks of the experiment. The doses were selected based on the literature data on the use flax oil, which is also characterized by a high ALA content. To determine the amount of oil administered, the BW of the rats was monitored every two days. The control rats (SHO; *n* = 10) received orally 1 mL of physiological saline. Animal plastic feeding tubes (Fuchigami, Kyoto, Japan) were used for the oral administration of oil or PhS. After 6 weeks of the experimental treatment, overnight fasted rats were weighted and euthanized by CO_2_ overdose. Then, blood was collected by cardiac puncture, and euthanasia was confirmed by cervical dislocation. Blood samples were collected into sterile tubes for clotting and then centrifuged at 3000 rpm for 30 min. Some serum samples were kept at −70 °C and others were stored at −20 °C until analysis. After euthanasia, the body composition, bone mineral density, and bone mineral content of total skeleton were determined. Afterwards, the right femora were dissected, cleaned of soft tissues, weighed, and frozen at −20 °C until further analysis.

### 2.2. Analysis of Fatty Acids in Camelina Oil

The fatty acids in the camelina oil were analyzed as methyl esters by gas chromatography. Fatty acid methyl esters (FAME) were prepared according to regulation No. PN-ISO 5509:1996 [36]. FAME analysis was performed using a gas chromatograph Agilent 6890N (Agilent Technologies, Santa Clara, CA, USA) equipped with a flame ionization detector and a HP-SSAP capillary column (30 m × 0.53 mm × 1 µm) according to PNEN ISO 5508:1996 [37]. The results for each fatty acid were expressed as % of total FAME. The composition of fatty acids in the camelina oil is presented in Table 1.

### 2.3. Densitometric Analysis (DXA) of the Body Composition, Whole Skeleton, and Isolated Femur

Bone mineral density (Tot.BMD) and bone mineral content (Tot.BMC) of the total skeleton and isolated right femora (fBMD, fBMC) were determined with the dual-energy X-ray absorptiometry method (DXA) using a Norland Excell Plus Densitometer (Norland, Fort Atkinson, WI, USA) equipped with Norland Illuminatus Small Subject Scan software v. 4.3.1. dedicated to small animals. The DXA method was also used for evaluation of the body composition parameters, e.g., lean mass (LM) and fat mass (FM). LM, FM, Tot.BMD, and Tot.BMC were measured immediately after euthanasia. Previously frozen femurs were kept at room temperature for 12 h before scanning. The scanning was performed using the following parameters: scout scan speed 100 mm/s, resolution 3.0 × 3.0 mm; measurement scan speed 10 mm/s, resolution 1.0 × 1.0 mm [38]. The region of interest (ROI) after the scout scan was defined manually. The densitometer was calibrated before every measurement series using quality assurance phantoms provided by the manufacturer in agreement with established procedures. To ensure credibility, all scans were performed by the same operator.

### 2.4. Peripheral Quantitative Computed Tomography (pQCT) of Isolated Femur

The right femurs were scanned by peripheral quantitative computed tomography (pQCT) using an XCT Research SA Plus system with software version 6.2.C (Stratec Medizintechnik GmbH, Pforzheim, Germany) [38]. The scans were performed in the middle diaphysis (at 50% of bone length) for the analysis of shaft microarchitecture and cortical bone tissue. Total bone mineral content (T.BMC), total volumetric bone mineral density (T.vBMD), total bone area (T.Ar), cortical bone area (Ct.Ar), cortical bone mineral content (Ct.BMC), cortical volumetric bone mineral density (Ct.vBMD), cortical thickness (Ct.Th), periosteal (Peri.C), and endocortical (Endo.C) circumferences, polar moment of inertia of cortical bone (PMI), and strength-strain index (SSI) were determined. Femur diaphysis was tested with the threshold set at 0.790 cm^−1^ and at cortical mode 2. Initial scan was performed at a speed of 10 mm/s, while proper scan was carried out at a speed of 4 mm/s. The pQCT system was calibrated before every measurement series with the use of the quality assurance phantom (pQCT QA-Phantom).

### 2.5. Mechanical Testing

After DXA and pQCT scanning, the mechanical parameters of isolated femurs were determined by a 3-point bending test with the use of a Zwick–Roell Testing Machine Z010 (Zwick–Roell GmbH and Co. KG, Ulm, Germany) as described earlier [38]. The apparatus was equipped with a measuring head (XForce HP series) with an operation range from 0 to 1 kN at a constant speed of 10 mm/min. After measuring the length, the bones were placed on two supports, and the measuring head loaded the midshaft perpendicularly to the horizontal axis at 50% of bone length. The distance between the supports was set at 40% of femur length. The data were analyzed with testXpert II 3.1 software (Zwick–Roell GmbH and Co. KG, Ulm, Germany). The ultimate force, work to fracture, and Young’s modulus were determined.

### 2.6. Markers of Bone Metabolism

Serum interleukin-6 (IL-6), osteocalcin, and C-terminal telopeptide of type I collagen (CTX-I) concentrations were measured using a commercial Rat IL-6 ELISA kit (DIACLONE SAS, Besançon, France, No. 670.010.096), a Rat MIDTM Osteocalcin EIA kit (Immunodiagnostic Systems Ltd., Boldon, Tyne and Wear, UK, No. AC-12F1), and a RatLaps CTX-ITM EIA kit (Immunodiagnostic Systems Ltd., Boldon, Tyne and Wear, UK, No. AC-06F1), respectively. The analysis of bone metabolism markers was performed using a Benchmark Plus microplate reader equipped with MicroplateManager software Version 5.2.1 (Bio-Rad Laboratories Inc., Hercules, CA, USA).

### 2.7. Statistical Aanalysis

All data were presented as mean values ± S.E.M. A one-way analysis of variance (ANOVA) and Tukey’s test were used to test the significant differences among the groups. For all comparisons, *p* < 0.05 was considered as statistically significant. The analysis of significant differences was performed with the use of STATISTICA 13.1 software (StatSoft, Inc. Tulsa, OK, USA).

## 3. Results

### 3.1. Body Weight, Body Composition, Bone Mass, and Length

The final BW and LM were significantly higher in the experimental rats than in the SHO group (Table 2). The BW in the CO-supplemented groups was by 10% higher than in the SHO group. There were no significant differences in BW and LM between the OVX-PhS and OVX rats treated with CO. The largest enhancement of LM was observed in the OVX-CO5 group (by 24% vs. SHO). Moreover, all experimental groups were characterized by higher values of FM, compared to the SHO animals. However, significant differences in comparison to the control animals were found in the OVX-CO9 group. The CO treatment significantly increased femur weight, compared to the SHO and OVX- PhS rats (Table 2).

### 3.2. DXA Parameters of Whole Skeleton and Femur

The values of the DXA parameters of the whole skeleton and isolated femurs are presented in Table 2. The ovariectomy significantly decreased total skeleton BMC and BMD by 12% and 8%, respectively, in comparison to these values in the SHO rats. Both groups receiving CO were characterized by higher Tot.BMC than the OVX-PhS group, i.e., by 12% and 18%, respectively. The values of this parameter were similar to those in the SHO group. Statistically significant differences in the Tot.BMD values were observed between the OVX-PhS and SHO groups. No significant differences in Tot.BMD were found between the SHO and CO-treated groups. The values of femur BMC and BMD were significantly lower in the OVX-PhS group than in the SHO and both OVX-CO groups, i.e., by 10%, 12%, and 11% for fBMC and by 3.5%, 7.2%, and 5.6% for fBMD, respectively. The values of fBMD and fBMC in the SHO and OVX-CO groups were similar.

### 3.3. pQCTMeasurements of Femur

The results of the pQCT measurement of cortical bone tissue in the femur midshaft are shown in Table 3. The lowest values of most of the analyzed parameters were observed in the OVX-PhS group. The camelina oil treatment significantly elevated T.vBMD, T.BMC, Ct.A, and Ct.Th, compared to these parameters in the OVX-PhS rats. The bone parameters were increased in the OVX-CO5 and OVX-CO9 groups as follows: T.vBMD—by 11.5% and 8%; T.BMC—by 8% and 9%; Ct.Ar—by 8% and 7%; and Ct.Th—by 10% and 8%, respectively. Moreover, Ct.BMC in the OVX-CO9 group was by 9% higher in comparison to the OVX-PhS group.

### 3.4. Mechanical Parameters of Femur

The results of the mechanical tests of bone are presented in Table 2. Although there were no statistically significant differences between the OVX-PhS and SHO rats, the values of the tested parameters were lower in the OVX rats in all cases. The ultimate force was significantly increased in the OVX-CO5 and OVX-CO9 groups in comparison to the OVX-PhS group. The values of ultimate force in the OVX-CO5 and OVX-CO9 groups were by 84% and 64% higher than in the OVX-PhS group. In turn, the values of work to fracture were also significantly higher in the OVX animals treated with CO than in those receiving PhS. Neither the ovariectomy nor the camelina oil treatment affected the Young’s modulus significantly.

### 3.5. Biochemical Markers of Bone Metabolism

The values of the biochemical markers of bone metabolism are presented in Table 4. The lowest serum osteocalcin concentration was noted in the control SHO rats. The ovariectomy caused an increase in the osteocalcin concentration. A significantly higher concentration of osteocalcin was observed in the CO-treated ovariectomized rats than in the SHO and OVX-PhS rats. The serum CTX-I concentration was significantly elevated in the OVX-PhS animals in comparison to the SHO and CO-treated groups. The serum concentration of CTX-I was by 63%, 36%, and 36% higher in OVX-PhS than in SHO, OVX-CO5, and OVX-CO9, respectively. The IL-6 concentration increased in the OVX-PhS animals compared with the SHO rats. No significant changes were observed between the CO-treated groups and the other groups. However, the concentration of IL-6 was lower in both CO groups than in the OVX-PhS animals.

## 4. Discussion

Ovariectomy results in deficient estrogen production and consequent negative changes in bone metabolism, leading to bone loss and increased susceptibility to fractures. However, a number of mechanisms may contribute of this effect. Ovary removal in rats also results in progressive bone loss and changes in bone parameters. Such changes in the densitometric, tomographic, and strength parameters were earlier found in experimental animals [39,40,41].

In the present study, the OVX rats exhibited significant bone loss manifested in the reduction in Tot.BMD, Tot.BMC, fBMD, fBMC, work to fracture, and T.vBMD. Moreover, the changes in the Il-6 and CTX-I levels detected in this study confirm the negative effect of ovariectomy on bone metabolism. In the OVX rats, the IL-6 and CTX-I levels were elevated in comparison to those in the control rats. Ovariectomy stimulates the IL-6 production, which is known to be up-regulated in a state of estrogen deficiency [42], and leads to an increase in the CTX-I concentration. Estrogen deficiency can also increase the concentration of IL-1, TNF-α [42], and other bone metabolism markers, i.e., bone alkaline phosphatase (bALP) as a marker of bone formation and bone disorders, as well as tartrate-resistant acid phosphatase (TRAP) as a bone resorption marker [43].

The results of our study indicate that the camelina oil treatment reduced the ovariectomy-stimulated negative changes in bone tissue. After the application of CO, we observed suppression in osteoclast-pathway activity, as evidenced by the suppression of the decrease in the values of Tot.BMC, T.vBMD, fBMD, fBMC, and work to fracture caused by estrogen deficiency. Moreover, the CO treatment restored the elevated level of the CTx values in the OVX rats to the SHO values. In a human study, a significant decrease in the CTX level was found after supplementation with n-3 PUFAs [44]. The decrease in bone resorption markers was also observed by Griel et al. [45]. In a study conducted by Boulbaroud et al. [43], flaxseed and sesame oil supplementation decreased bALP and TRAP levels. However, there are also literature data that indicate no changes in bone resorption and formation markers in healthy individuals [46].

Based on the present results, it can be assumed that CO also affects bone formation. This is evidenced by the higher values of some parameters in the OVX oil-receiving animals compared to the SHO group. Primarily, the OVX rats receiving CO had significantly higher serum osteocalcin levels than the SHO animals. This increase in the osteocalcin concentration may evidence bone turnover on the one hand and bone formation on the other. However, the simultaneous increase in the values of bone mass, Ct.Th, Ct.BMC, and T.vBMD indicates stimulation of bone formation.

Previous studies have shown that n-3 PUFAs supplementation reduced bone loss in OVX rats [47,48]. Moreover, a positive effect of dietary soybean, as well as flaxseed and sesame oils, on femur bone density and calcium content in bone ash was observed in osteoporotic rats by Wahba et al. [9]. In turn, a positive effect of flaxseed and sesame oils connected with partial mitigation of osteoporotic changes in OVX rats was described by Boulbaroud et al. [43]. A beneficial impact of flaxseed oil and soybean oil on the prevention of negative changes in bone rats was also reported by Hassan et al. [10] and El-Saeed et al. [13]. As shown by Elbahnasawy et al. [14], enhanced bone formation and reduction in bone loss markers, as reflected by reduced CTX levels in plasma, was observed after administration of soybean and flaxseed oils in a GC-induced osteoporosis model. Thus, our results are in line with other studies.

There are several mechanisms by which n-3 and n-6 fatty acids affect bone tissue. For instance, PUFAs can modulate calcium absorption, production of inflammatory cytokine and prostaglandins, and regulation of osteoblast and osteoclast activity by changing the fatty acid composition in bone cell membranes [49,50]. n-3 PUFAs have been reported to suppress osteoclast activity and improve osteoblast activity [51,52]. PUFAs influence intestinal calcium absorption via both active and passive transport. DHA, but not EPA and AA, stimulated active calcium transport by increasing Ca^2+^-ATPase activity in the basolateral membrane of duodenal enterocytes [53]. However, supplementation with fish oil and evening primrose oil also resulted in an increase in calcium transport across the basolateral membrane [54]. The changes in calcium transport are caused by the incorporation of long-chain PUFAs into the cell membrane. On the one hand, this leads to increased membrane fluidity, permeability, and speed of flip-flop transport across the membrane and, in consequence, cellular uptake of different molecules, particularly via passive transport [55,56]. On the other hand, increased membrane unsaturation alters the dispersion of lipid rafts within membranes and can modulate the activity of membrane proteins, e.g., Ca^2+^-ATPase [57,58].

Inflammatory cytokines play a crucial role in the pathogenesis of osteoporosis by stimulation of bone resorption and inhibition of osteosynthesis. IL-1, IL-6, and TNF-α are involved in bone metabolism [8]. n-6 PUFAs increase the synthesis of proinflammatory cytokines by bone cells, whereas n-3 acids attenuate this effect [45,59]. Upon increasing dietary intake of n-3 PUFAs, n-3 acids partially replace n-6 acids in the cell membranes, and the increasing n-3/n-6 PUFA ratio may result in changes in membrane function, and reduction in the synthesis of proinflammatory factors [60,61,62]. Flaxseed oil supplementation was reported to reduce the TNF-α level [45,63]. In turn, no changes in IL-1 and IL-6 levels after flaxseed and walnut oil supplementation were observed [45]. In our study, we investigated the serum level of IL-6, which was elevated by the ovariectomy. The camelina oil supplementation reversed this effect. However, these results were not statistically significant. Il-6 is known to promote the development of osteoclasts and to stimulate bone resorption [8]. Its production was stimulated by 2-series prostaglandins (PGE_2_) [64,65] and Il-1 [66]. PGE_2_ is one of the modulators of bone remodeling. It has been shown to mediate both bone formation and resorption [67,68]. At a physiological concentration, PGE_2_ positively influences bone growth [69,70].

Modification of dietary PUFAs leads to alterations in prostaglandin metabolism [71,72,73]. PUFAs n-6 and n-3 are precursors for PGE_2_ and PGE_3_, respectively. Dietary PUFAs, particularly the n-6/n-3 ratio, are important factors for the bone marrow fatty acid profile, and this in turn determines the capacity of bone for synthesis of PGE_2_ [72,73,74]. It was found that a rise in the dietary n-6/n-3 ratio was accompanied by an increase in the level of PGE_2_ and a decline in Ca, P, and Mg contents in bone [73]. On the other hand, a low ratio of n-6/n-3 PUFAs decreased PGE_2_ production and improved bone formation [69]. AA, cleaved from membrane phospholipids of osteoblasts, is a substrate of cyclooxygenase-2 (COX-2) for PGE_2_ synthesis. Some data indicate that n-6 PUFAs up-regulate COX-2 expression and stimulate production of PGE_2_, whereas n-3 PUFAs diminish COX-2 expression [75]. An increase in the n-3 PUFAs content in diet may lead to displacement of AA from phospholipids and reduction in PGE_2_ synthesis [71,76]. PGE_3_ synthesized from EPA also mediates bone cell function. However, AA is more easily synthesized to PGE_2_ than EPA is to PGE_3_ [77]. The reduction in PGE_2_ was observed in rats after flaxseed and menhaden oil administration, but it was not accompanied by changes in femur area, BMC, and BMD [72]. Similar results were observed in chicken receiving flaxseed oil alone or in combination with palm oil [78]. The decreased PGE_2_ concentration was not associated with changes in bone characteristics. However, flaxseed oil supplementation led to an increase in the ALP concentration and a decrease in the TRAP concentration.

A high level of PGE_2_ leads to bone resorption [79,80]. This may be related to stimulation of the expression of the receptor activator of nuclear factor NF-kB ligand (RANKL) on osteoblast and its receptor (receptor activator of nuclear factor NF-kB, RANK) on osteoclasts and reduction in the expression of the decoy receptor, osteoprotegerin (OPG), on osteoblasts by the high level of PGE_2_ [81,82]. The binding of RANKL to OPG prevents osteoclastogenesis, whereas binding to RANK stimulates this process [83]. Currently, the balance of RANKL/OPG is considered very important for bone metabolism and the pathogenesis of resorptive bone disease [84]. Thus, a high level of PGE_2_ has a stimulating effect on osteoclastogenesis and OC activity [85,86]. It has been found that AA leads to enhanced synthesis of RANKL, which binds to RANK on osteoclast precursors stimulating osteoclastogenesis and maturation of osteoclasts [79]. Moreover, AA inhibits the synthesis of OPG [82]. PGE_3_ synthesized from EPA also promotes osteoclastogenesis but the conversion EPA to PGE_3_, as mentioned before, is less efficient.

The favorable effect of n-3 acids on bone tissue was observed not only in rats, but also in other animal species [87]. Higher values of the cortical wall thickness, cross-sectional area, cortical index, BMC, BMD, maximum elastic strength, and maximum strength were determined for femurs of pigs after linseed oil supplementation [88]. Flax oil alone or in combination with palm oil had a positive effect on the biomarkers of bone growth and increased tibia calcium levels in broiler chickens [78]. However, no significant differences in BMD, BMC, strength parameters, and ash percentage after flaxseed supplementation were observed in piglets and chickens [15,89,90]. The source of PUFAs, i.e., the type of oil, seems to be important for the impact of PUFAs on bone tissue. Equally important is the ratio of n-3 to n-6 PUFAs. Nevertheless, the literature data on the effect of individual vegetable oils on animal bones are not explicit.

## 5. Conclusions

Our research has shown that camelina oil has a positive osteotropic effect via inhibition of adverse changes in bones caused by ovariectomy. The administration of the oil yielded similar or even better densitometric, tomographic, and mechanical parameters of the bones in the OVX rats in comparison with the values in the control rats. These results are confirmed by the changes in the concentration of bone metabolism markers. The decrease in the serum concentration of CTX-I with the simultaneous increase in the concentration of osteocalcin indicates suppression of bone tissue resorption by inhibition of the osteoclastic-pathway on the one hand and an increase in bone turnover with a simultaneous advantage of bone formation over resorption on the other hand. Camelina oil supplementation can be an efficient method for improving bone health in a disturbed health state. However, further research must be carried out on other animal species supplemented with this oil. In addition, it is also necessary to investigate the effect of camelina oil on bones in individuals without bone changes.

## Figures and Tables

**Table 1:** Fatty acid composition of camelina oil. The contents of particular fatty acids are presented as a percentage of total FAME.

Fatty Acids	Content
C14:0	0.05
C15:0	0.02
C16:0	5.12
C16:1	0.13
C17:0	0.06
C18:0	2.42
C18:1	14.94
C18:2	17.42
C18:3	33.95
C20:0	1.35
C20:1	14.35
C20:2	1.44
C20:4	1.03
C22:0	0.3
C22:1	2.91
C24:0	0.18
C24:1	0.67
Total n-6 PUFA	19.89
Total n-3 PUFA	33.95

**Table 2:** Final body weight, parameters of body composition, and densitometric and mechanical properties of femur.

Parameters	SHO	OVX-PhS	OVX-CO5	OVX-CO9
BW (g)	295 ± 2 ^a^	305 ± 6	324 ± 7 ^b^	324 ± 10 ^b^
LM (g)	213 ± 5 ^a^	258 ± 8 ^b^	264 ± 7 ^b^	247 ± 6 ^b^
FM (g)	23.8 ± 2.4 ^a^	36.6 ± 5.8	47.1 ± 7,0	58.0 ± 11.9 ^b^
Tot.BMC (g)	8.58 ± 0.20 ^a^	7.55 ± 0.11 ^b^	8.49 ± 0.24 ^a^	8.94 ± 0.35 ^a^
Tot.BMD (g/cm^2^)	0.143 ± 0.003 ^a^	0.133 ± 0.002 ^b^	0.137 ± 0.001	0.136 ± 0.001
Femur				
weight (g)	0.78 ± 0.05 ^a^	0.77 ± 0.04 ^a^	0.85 ± 0.06 ^b^	0.89 ± 0.08 ^b^
length (mm)	34.9 ± 0.05	34.8 ± 0.06	35.3 ± 0.03	35.1 ± 0.06
fBMD (g/cm^2^)	0.094 ± 0.002 ^a^	0.091 ± 0.001 ^b^	0.098 ± 0.002 ^a^	0.096 ± 0.001 ^a^
fBMC (g)	0.336 ± 0.010 ^a^	0.301 ± 0.006 ^b^	0.341 ± 0.008 ^a^	0.338 ± 0.007 ^a^
Ultimate force (N)	111.3 ± 7.3 ^a^	96.4 ± 7.1 ^a^	176.9 ± 13.2 ^b^	157.8 ± 12.3 ^b^
Work to fracture (mJ)	14.35 ± 2.08 ^ac^	10.86 ± 2.77 ^a^	37.75 ± 6.33 ^b^	29.82 ± 4.94 ^bc^
Young’s modulus (GPa)	3.38 ± 0.15	2.79 ± 0.13	3.43 ± 0.39	3.03 ± 0.34

The results are means ± S.E.M (*n* = 10). The values in the rows are significantly different (*p* ≤ 0.05) as indicated by the superscripts: a, b, c. Abbreviations: SHO —shame operated rats receiving physiological saline; OVX-PhS —ovariectomized rats receiving physiological saline; OVX-CO5 —ovariectomized rats receiving camelina oil in the amount of 5 g/kg/BW; OVX-CO9 —ovariectomized rats receiving camelina oil in the amount of 9 g/kg/BW; BW—body weight; LM—lean mass; FM—fat mass; Tot. BMC—bone mineral content of total skeleton; Tot. BMD—bone mineral density of total skeleton; fBMD—bone mineral density of femur; fBMC—bone mineral content of femur.

**Table 3:** Tomographic analysis (pQCT) of bone tissue in the middle part of femur diaphysis.

Parameters	SHO	OVX-PhS	OVX-CO5	OVX-CO9
T.vBMD (mg/mm^3^)	805 ± 6 ^a^	764 ± 9 ^b^	852 ± 7 ^c^	827 ± 6 ^ac^
T.BMC (mg/mm)	7.30 ± 0.11	7.15 ± 0.04 ^a^	7.74 ± 0.19 ^b^	7.81 ± 0.18 ^b^
Ct.vBMD (mg/mm^3^)	1420 ± 4	1419 ± 3	1424 ± 5	1430 ± 4
Ct.BMC (mg/mm)	6.83 ± 0.12 ^a^	6.74 ± 0.06 ^a^	7.19 ± 0.17	7.32 ± 0.14 ^b^
T.Ar (mm^2^)	9.09 ± 0.18	9.08 ± 0.19	9.31 ± 0.23	9.69 ± 0.17
Ct.Ar (mm^2^)	4.73 ± 0.07	4.77 ± 0.06 ^a^	5.13 ± 0.11 ^b^	5.11 ± 0.09 ^b^
Ct.Th (mm)	0.527 ± 0.003 ^a^	0.522 ± 0.004 ^a^	0.569 ± 0.011 ^b^	0.558 ± 0.006 ^b^
Peri.C (mm)	10.7 ± 0.1	10.7 ± 0.1	10.8 ± 0.1	10.9 ± 0.1
Endo.C (mm)	7.41 ± 0.10	6.89 ± 0.64	7.37 ± 0.15	7.46 ± 0.10
PMI (mm^4^)	10.35 ± 0.37	10.17 ± 0.33	11.24 ± 0.45	11.58 ± 0.42
SSI (mm^3^)	5.67 ± 0.12	5.45 ± 0.10	5.97 ± 0.19	6.17 ± 0.22

The results are means ± S.E.M (*n* = 10). The values in the rows are significantly different (*p* ≤ 0.05) as indicated by the superscripts: a, b, c. Abbreviations: T.vBMD—total volumetric bone mineral density; T.BMC—total bone mineral content; Ct.vBMD—cortical volumetric bone mineral density; Ct.BMC—cortical bone mineral content; T.Ar—total bone area; Ct.Ar—cortical bone area; Ct.Th—cortical thickness; Peri.C—periosteal circumferences; Endo.C—endocortical circumferences; PMI—polar moment of inertia of the cortical bone; SSI—strength-strain index.

**Table 4:** Biochemical markers of bone metabolism.

Parameters	SHO	OVX-PhS	OVX-CO5	OVX-CO9
Osteocalcin (ng/mL)	129.7 ± 8.4 ^a^	246.9 ± 14.8 ^b^	378.2 ± 25.1 ^c^	356.7 ± 23.0 ^c^
CTX-I (ng/mL)	18.37 ± 1.95 ^a^	30.01 ± 2.11 ^b^	22.13 ± 1.68 ^a^	22.14 ± 1.74 ^a^
IL-6 (pg/mL)	280.3 ± 26.2 ^a^	457.2 ± 53.7 ^b^	307.5 ± 32.1	292.7 ± 39.9

The results are means ± S.E.M (*n* = 10). The values in the rows are significantly different (*p* ≤ 0.05) as indicated by the superscripts: a, b, c. Abbreviations: CTX-1—C-terminal telopeptide of type I collagen; IL-6—interleukin-6.

## Data Availability

The data presented in this study are available on request from the corresponding author.
